# Predicting lncRNA-disease associations using multiple metapaths in hierarchical graph attention networks

**DOI:** 10.1186/s12859-024-05672-2

**Published:** 2024-01-29

**Authors:** Dengju Yao, Yuexiao Deng, Xiaojuan Zhan, Xiaorong Zhan

**Affiliations:** 1 School of Computer Science and Technology, Harbin University of Science and Technology, Harbin, 150080 China; 2 College of Computer Science and Technology, Heilongjiang Institute of Technology, Harbin, 150050 China; 3 Department of Endocrinology and Metabolism, Hospital of South, University of Science and Technology, Shenzhen, 518055 China

**Keywords:** Metapaths, Heterogeneous graph, Multihead attention mechanism, lncRNA-disease associations

## Abstract

**Background:**

Many biological studies have shown that lncRNAs regulate the expression of epigenetically related genes. The study of lncRNAs has helped to deepen our understanding of the pathogenesis of complex diseases at the molecular level. Due to the large number of lncRNAs and the complex and time-consuming nature of biological experiments, applying computer techniques to predict potential lncRNA-disease associations is very effective. To explore information between complex network structures, existing methods rely mainly on lncRNA and disease information. Metapaths have been applied to network models as an effective method for exploring information in heterogeneous graphs. However, existing methods are dominated by lncRNAs or disease nodes and tend to ignore the paths provided by intermediate nodes.

**Methods:**

We propose a deep learning model based on hierarchical graphical attention networks to predict unknown lncRNA-disease associations using multiple types of metapaths to extract features. We have named this model the MMHGAN. First, the model constructs a lncRNA-disease–miRNA heterogeneous graph based on known associations and two homogeneous graphs of lncRNAs and diseases. Second, for homogeneous graphs, the features of neighboring nodes are aggregated using a multihead attention mechanism. Third, for the heterogeneous graph, metapaths of different intermediate nodes are selected to construct subgraphs, and the importance of different types of metapaths is calculated and aggregated to obtain the final embedded features. Finally, the features are reconstructed using a fully connected layer to obtain the prediction results.

**Results:**

We used a fivefold cross-validation method and obtained an average AUC value of 96.07% and an average AUPR value of 93.23%. Additionally, ablation experiments demonstrated the role of homogeneous graphs and different intermediate node path weights. In addition, we studied lung cancer, esophageal carcinoma, and breast cancer. Among the 15 lncRNAs associated with these diseases, 15, 12, and 14 lncRNAs were validated by the lncRNA Disease Database and the Lnc2Cancer Database, respectively.

**Conclusion:**

We compared the MMHGAN model with six existing models with better performance, and the case study demonstrated that the model was effective in predicting the correlation between potential lncRNAs and diseases.

## Introduction

LncRNAs can regulate the expression of target genes through different cellular mechanisms, such as signal transduction, induction, guidance, and scaffolding, and play a variety of roles in all life processes [1]. Aberrant expression of lncRNAs is usually associated with human diseases. Therefore, mining the correlation between lncRNAs and diseases is conducive to elucidating the pathogenic mechanisms of complex diseases, providing a basis for disease diagnosis and prevention.

Although some lncRNA-disease associations have been experimentally validated, the vast majority of these associations remain unknown [1]. Traditional biological experimental approaches to validate potential lncRNA-disease associations are often resource intensive and costly. To alleviate this problem, computational approaches have received much attention from scholars. Recent methods can be broadly classified into three categories: network-based methods, random walk-based methods, and machine learning-based methods.

Network-based approaches focus on predicting potential associations between lncRNAs and diseases using various propagation algorithms. The first network-based method, LRLSLDA [2], combines the lncRNA-disease association network and the lncRNA expression similarity network and incorporates Laplace's regular least squares in a semisupervised learning framework to identify potential lncRNA-disease associations. Notably, this approach does not require negative samples. Yang et al. [3] used a propagation algorithm to identify existing diseases and detected disease-causing gene associations; based on this information, they constructed a new disease gene-related network and identified lncRNA-disease associations in that network. Li [4] calculated multiple similarities between lncRNAs and diseases, acquired probability matrices of lncRNAs and diseases, and subsequently assessed their network consistency before predicting unknown lncRNA-disease associations. Zhang et al. [5] combined lncRNA, protein, and disease information to construct a network and applied the stream propagation algorithm.

Random walk-based methods can pay more attention to the information that contributes more to the network. Xie et al. [6] proposed the LDA-LLNSUBRW model to predict LDA. This model is based mainly on linear neighborhood similarity and an unbalanced bi-random walk. Sun et al. [7] proposed the RWRlncD method, which is based on a global network that contains the lncRNA functional similarity network, the disease similarity network, and known lncRNA-disease associations. For lncRNAs without a known associated disease, however, this approach cannot be applied. Li et al. [8] designed an improved local random walk method for a newly established heterogeneous network. In 2019, Hu et al. [9] introduced a matrix completion method (LMNLMI).

The third category includes machine learning-based methods. Yao et al. [10] utilized random forests to select features in their proposed methodology. Wang et al. [11] proposed a weighted matrix decomposition (WMD) method for LDA prediction by presetting the weights of different correlation matrices and converting them into low-dimensional matrices. Lan et al. [12] trained a support vector machine (SVM) model to predict potential associations between lncRNAs and diseases by combining multiple biological data. Yu et al. [13] created a predictive model (CFNBC) based on Bayesian classification by unifying the associations among lncRNAs, diseases, and miRNAs. Bayesian classification was used for linear discriminant analysis (LDA) prediction models of collaborative filtering (CFNBC). With the growth of scientific research, there has been an increasing emphasis on neural networks. Neural networks can achieve superior training results by continuously modifying parameters through numerous operations. Recently, graphical neural networks, such as graphical convolutional networks (GCNs) and graphical attention networks (GATs), have been used in bioinformatics research because of their ability to integrate graph topology and node features. To prioritize more relevant neighbors and eliminate noise, they have also developed a bi-interaction aggregator to aggregate representations of similar neighbors. The GBDT-LR [14] model uses two different machine learning methods, gradient boosting decision trees and logistic regression, and combines them. Wu et al. [15] developed the GAMCLDA model, which applies graph convolutional networks to reconstruct graph structures and lncRNA and disease node feature vectors.

These existing methods have achieved satisfactory performance and effectively contributed to the advancement of computational methods for LDA prediction, but the ability of these methods to mine the rich semantic information in heterogeneous graphs composed of lncRNAs and diseases is far from optimal or even satisfactory. Metapaths show strong potential for exploring complex structural and semantic information in heterogeneous networks. Xuan [16] et al. considered that nodes with similar attributes are not only located near the neighborhood of the target node but also located in the region far from the target node. Therefore, they integrated the associations between the nodes, increased global dependencies, and added multiview features of the node pairs. Zhao [17] et al. developed a new framework based on heterogeneous graph attention networks and metapath graph attention networks. They constructed a two-part topological graph of lncRNAs and diseases and used the KNN algorithm to remove noise effects. Inspired by existing studies, we designed a multiple metapath-based hierarchical graph attention network model for lncRNA-disease association prediction. The approach of constructing subgraph aggregation features under multiple types of metapaths is used to obtain information about various relationships between lncRNAs and diseases in both heterogeneous and homogeneous graphs simultaneously for better performance. Our contributions are as follows:

1. We propose a dual-path feature extraction strategy based on a homogeneous graph and a heterogeneous graph. Subgraph aggregation features of homomorphic and heteromorphic graphs are used to enrich the model input information. The KNN algorithm is used to construct homogeneous subgraphs to reduce computation and denoising. In addition, miRNA information nodes are introduced to construct a ternary heterogeneous network with richer information.
2. Different types of metapaths are constructed. For the heterogeneous graph, the existing metapaths are only paths for lncRNA or disease nodes, i.e., the connecting pathways of other nodes, such as miRNA nodes, are ignored. We learn each homogeneous graph or heterogeneous subgraph of a specific metapath by extracting the paths that lncRNAs or disease nodes reach through different types of nodes using the GAT network. Moreover, in the heterogeneous subgraphs, we adaptively assign weights to the different metapath subgraphs using the attention mechanism to obtain additional semantic information.

## Materials and methods

### Datasets

In this study, datasets collected from three studies were used to evaluate the model performance.

Dataset 1: The dataset used in the study by Fu [18] is widely used as a reliable dataset. The main sources are Lnc2Cancer [19], LncRNADisease [20], GeneRIF [21], and starBase v2.0 [22] HMDD v2.0 [23].

Dataset 2: We referred to the dataset screened in Zhou's [24] study. The lncRNAs were integrated from the lncr2cancer v3.0 [25], LncRNADisease v2.0 [26], starBase v2.0 [22] and HMDDv3.2 databases.

Dataset 3: We used the dataset screened by Li et al. [27]. The authors screened relevant records with causal relationships from the HMDDV3.2 database and converted all disease names into standardized names based on the MeSH nomenclature. Finally, 861 lncRNAs, 437 miRNAs, and 432 diseases were obtained.

Model parameter tuning, ablation experiments, and comparisons with the baseline model were performed on dataset 1. Three datasets were used for robustness experiments. The detailed data are shown in Table 1. In this table, LDA represents the association of lncRNAs with diseases, LMA represents the association of lncRNAs with miRNAs, and MDA represents the association of miRNAs with diseases.

**Table 1:** Dataset information

Dataset	lncRNA	Disease	MiRNA	LDA	LMA	MDA
Dataset 1	240	412	495	2697	1002	13,562
Dataset 2	665	316	295	3833	2108	8540
Dataset 3	861	432	437	4516	8166	4189

### Flowchart of the MMHGAN model

**Fig. 1:**
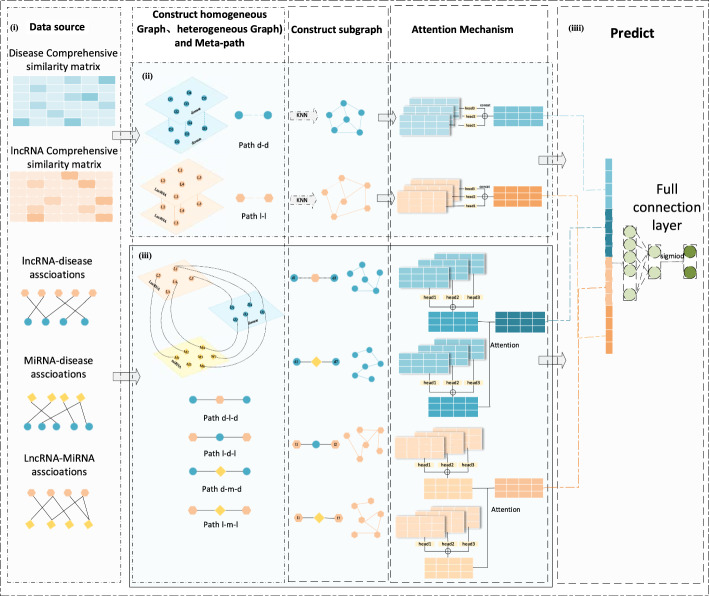
Flowchart of the MMHGAN model. The MMHGAN model consists of four stages. (i) Calculate the combined similarity between lncRNAs and diseases and collate the associations between lncRNAs and diseases and between miRNAs. (ii) Construct homogeneous graphs GL and GD based on the top k pieces of information with the highest similarity in the combined similarity matrix of lncRNAs and diseases derived from the KNN algorithm. Aggregated the neighbor node features through the multihead attention mechanism. (iii) Construct a heterogeneous graph G_lmd_ based on the association matrix, extract different types of metapaths from the graph, construct subgraphs, and update node embeddings through a graph attention network (GAT). Subsequently, calculated the weights under different metapaths and update the target node embeddings. (iv) Use the fully connected layer to recombine the input features to predict potential lncRNA-disease associations

As shown in Fig. 1, we propose the MMHGAN model for predicting lncRNA candidates associated with a given disease. The MMHGAN model consists of data sources, the construction of heterogeneous and homogeneous graphs, the acquisition of subgraph features via multihead attention, and prediction.

### LncRNA sequence similarity

We obtained the sequences by lncRNA name from NONCODE (http://www.noncode.org/), GenBank (https://www.ncbi.nlm.nih.gov/) and Ensembl (http://asia.ensembl.org/index.html) to obtain information to find the corresponding sequence of each lncRNA. After obtaining all the lncRNA sequences, based on previous studies by Yang [28] and Li [29] et al., we performed a two-by-two calculation of the lncRNA sequences using the Levenshtein distance, which is the editing distance between strings used to measure the differences between two strings [30]. In previous studies, the editing cost was set to 2, while the insertion cost and deletion cost were set to 1. We followed the same criteria in our study. The formula for the LSS is shown below:

1 $$LSS\left({l}_{i},{l}_{j}\right)=1-\frac{dist}{len({l}_{i}+{l}_{j})}$$

where dist denotes the minimum cost of converting the ${l}_{i}$ sequence of a lncRNA to the ${l}_{j}$ sequence and len denotes the length of the lncRNA sequence.

### Disease semantic similarity

The computation of the semantic similarity of diseases is based on the medical subject term descriptor [31], available from https://www.ncbi.nlm.nih.gov/. [32] The tool provides topological relationships between diseases and describes them with a directed acyclic graph (DAG). With the known directed acyclic graph, we calculated the semantic similarity DSS between diseases using the method proposed by Wang et al. [32]. Assuming that d is an ancestor node of the DAG and ${d}{\prime}$ is a child node of d, the semantic contribution of each node in the DAG is calculated as follows:

2 $$\left\{\begin{array}{l}{D}_{D1}\left(d\right)=1 \qquad if\; d=D\\ {D}_{D1}\left(d\right)={\text{max}}\left\{0.5\times {D}_{D1}\left({d}{^{\prime}}\right)|{d}{^{\prime}}\epsilon children\; of\; d\right\} \qquad if \,\,d\ne D\end{array}\right.$$

After the contribution scores were obtained, the semantic score ${D}_{v1}$ was calculated for each disease:

3 $${D}_{V1}\left(D\right)=\sum_{d\in T(D)}{D}_{D1}(d)$$

T represents the DAG topology of the disease.

Finally, the semantic similarity of the two diseases was calculated with the following formula:

4 $$DSS(d(i),d(j))=\frac{{\sum }_{t\in T(d(i))\cap T(d(j)}({D}_{d(i)}(t)+{D}_{d(j)}(t))}{DV(d(i))+DV(d(j))}$$

### LncRNA/disease GIP kernel similarity

According to previous studies, the lncRNA Gaussian kernel similarity (LGS) and disease Gaussian kernel similarity (DGS) were calculated based on the neighbor-joining matrix LD. The formula for the LGS is as follows:

5 $$LGS\left({l}_{i},{l}_{j}\right)={\text{exp}}(-{\xi }_{l}{\Vert LD\left(i,:\right)-LD(j,:)\Vert }^{2})$$

6 $${\xi }_{l}=1/\left(\frac{1}{{N}_{l}}\sum_{i=1}^{{N}_{l}}{\Vert LD(i,:)\Vert }^{2}\right)$$

Here, ${N}_{l}$ denotes the number of lncRNAs, and ${\xi }_{l}$ is the regularization factor.

Similarly, the DGS was calculated as follows:

7 $$DGS\left({d}_{i},{d}_{j}\right)={\text{exp}}(-{\xi }_{d}{\Vert LD\left(i,:\right)-LD(j,:)\Vert }^{2})$$

8 $${\xi }_{d}=1/\left(\frac{1}{{N}_{d}}\sum_{i=1}^{{N}_{d}}{\Vert LD(i,:)\Vert }^{2}\right)$$

Here, ${N}_{d}$ denotes the number of diseases, and ${\xi }_{d}$ is the regularization factor.

Considering that there are many sparse values in the similarity matrix obtained above and that there is a problem with inaccurate prediction of individual semantic information as features, we linearly fused the two similarities in the following equation:

9 $$LSM=\frac{\alpha LSS\left({l}_{i},{l}_{j}\right)+\left(1-\alpha \right)LGS\left({l}_{i},{l}_{j}\right)}{2}$$

10 $$DSM=\frac{\alpha DSS\left({l}_{i},{l}_{j}\right)+(1-\alpha )DGS({l}_{i},{l}_{j})}{2}$$

LSM and DSM are the combined similarity matrices of lncRNAs and disease after linear fusion.

### Feature extraction based on heterogeneous graphs

#### Subgraph construction based on metapaths

A metapath is a composite relation connecting two objects and is a widely used structure for capturing semantics. Metapaths can be used to explore structural information in heterogeneous graphs and capture rich semantic information, fully and intuitively exploiting network structures.

To explore more diverse information embedded in the metapaths, we constructed a ternary heterogeneous graph ${G}_{lmd}=(V,E)$ containing three types of nodes, lncRNA, miRNA, and disease nodes. The set of nodes is $v=\left\{{v}^{lnc}\cup {v}^{dis}\cup {v}^{mir}\right\}$. ${v}^{lnc}$ represents the set of 240 lncRNA nodes, ${v}^{dis}$ is the set of 412 disease nodes, and ${v}^{mir}$ is the set containing 495 miRNA nodes. The edge E in the heterogeneous graph can be defined as follows:

11 $$E=\left\{\begin{array}{c}{E}^{lnc-dis}\in {R}^{{N}_{lnc}\times {N}_{dis}}\\ {E}^{lnc-mir}\in {R}^{{N}_{lnc}\times {N}_{mir}}\\ { E}^{mir-dis}\in {R}^{{N}_{mir}\times {N}_{dis}}\end{array}\right.$$

where ${N}_{lnc}$, ${N}_{dis}$ and ${N}_{mir}$ represent the numbers of lncRNAs, diseases and miRNAs in the dataset, respectively. ${E}^{lnc-dis}$, ${E}^{lnc-mir}$ and ${E}^{mir-dis}$ represent the association matrix of lncRNAs and diseases, the association matrix of lncRNAs and miRNAs and the association matrix of miRNAs and diseases, respectively. Given lncRNA node ${l}_{i}$ $({l}_{i}\in {N}_{lnc})$ and disease node ${d}_{j} ({d}_{j}\in {N}_{dis})$, there is an association between ${l}_{i}$ and ${d}_{j}$ if the association matrix ${E}_{ij}^{lnc-dis}=1$. If ${E}_{ij}^{lnc-dis}=0$, then an association between ${l}_{i}$ $({l}_{i}\in {N}_{lnc})$ and ${d}_{j} ({d}_{j}\in {N}_{dis})$ has not yet been observed. Similarly, if ${E}_{ij}^{lnc-mir}=1$ or ${E}_{ij}^{mir-dis}=1$, nodes ${l}_{i}$ $({l}_{i}\in {N}_{lnc})$ and ${m}_{j} ({m}_{j}\in {N}_{mir})$ or ${m}_{i}$ $({l}_{i}\in {N}_{mir})$ and disease node ${d}_{j} ({d}_{j}\in {N}_{dis})$ are associated; otherwise, ${E}_{ij}^{lnc-mir}=0$ or ${E}_{ij}^{mir-dis}=0$.

The correlation matrix G between the heterogeneous maps ${G}_{lmd}$ can be defined as:

12 $$G=\left[\begin{array}{ccc}0& {E}^{lnc-dis}& {E}^{lnc-mir}\\ {{E}^{lnc-dis}}^{T}& 0& { E}^{mir-dis}\\ {{E}^{lnc-mir}}^{T}& {{ E}^{mir-dis}}^{T}& 0\end{array}\right]$$

Dataset 1 was chosen as an example, and 2697 lncRNA-disease associations were experimentally verified. We treated these 2697 experimentally verified associations as positive samples, labeled 1. However, the number of known lncRNA-disease associations is much greater than the number of known lncRNA-disease associations. An imbalance of positive and negative samples reduces the generalizability of the model. To address this issue, we randomly selected an equal number of unknown lncRNA-disease associations, labeled 0, to be added to the heterogeneous map. In addition, we used the combined similarity of lncRNAs, miRNAs, and diseases as lncRNA and disease node features, respectively. Therefore, the lncRNA node feature has 240 dimensions, the disease node feature has 412 dimensions, and the feature vector is represented as a lncRNA, for example:

13 $${F}_{li}=\left({x}_{1};{x}_{2};{x}_{3};\dots \dots ;{x}_{239},{x}_{240}\right)$$

14 $${F}_{di}=({y}_{1};{y}_{2};{y}_{3}\dots \dots ;{y}_{241},{y}_{412})$$

where ${F}_{li}$ represents the features of the ith lncRNA in the lncRNA similarity matrix and ${x}_{j}$ represents the combined similarity value of the ith lncRNA and the jth lncRNA. Similarly, ${F}_{di}$ represents the feature vector of the ith disease in the disease similarity matrix.

Pathways essentially describe the associations between lncRNAs ${L}_{1}$ and ${L}_{2}$ or between diseases ${D}_{1}$ and ${D}_{2}$. Different metapaths usually have different semantics. In the ternary heterogeneous graph ${G}_{lmd}$ obtained above, it is assumed that there is a metapath type P of $L1\to D1\to L2$, ${L}_{1}$ is a certain lncRNA node, ${D}_{1}$ is a certain disease node with which it is associated, and ${L}_{2}$ is another lncRNA associated with the above disease node. Through the metapath p, if there exists a node v that conforms to the metapath type P, then the set of nodes ${v}_{l}^{pD}$ can be obtained. Thus, we can obtain the subgraph ${G}_{l}^{pD}=({v}_{l}^{pD},{E}_{ld})$ of the LncRNA. ${E}_{ld}$ represents the edges formed by lncRNA nodes conforming to the metapath connections of a given type. In our proposed model, in addition to the metapaths of type $L\to D\to L$, we define three other types of metapaths $L\to M\to L$, $D\to L\to D$, and、$D\to M\to D$. With these three types of metapaths, we can construct the following three kinds of homogeneous subgraphs:

${G}_{l}^{pM}=({v}_{l}^{pM},{E}_{lm})$. ${v}_{l}^{pM}$ represents the set of lncRNA nodes for which a metapath type PM exists for lncRNA nodes, and ${E}_{lm}$ represents the edges formed by connecting lncRNA nodes through miRNA nodes.

${G}_{d}^{pM}=({v}_{d}^{pM},{E}_{dm})$. ${v}_{d}^{pM}$ represents the set of disease nodes for which a metapath type PM exists for disease nodes, and ${E}_{dm}$ represents the edges formed by connecting disease nodes through miRNA nodes.

${G}_{d}^{pL}=({v}_{d}^{pL},{E}_{dl})$. ${v}_{d}^{pL}$ represents the set of disease nodes for which a metapath type PL exists for disease nodes, and ${E}_{dl}$ represents the edges formed by connecting disease nodes through lncRNA nodes.

### Feature extraction

After obtaining the above homogeneous subgraph, different nodes were found to be in different feature spaces due to the heterogeneity of nodes in the lncRNA-disease–miRNA heterogeneity graph. To address feature nodes in the same space, we performed a linear transformation on the three types of nodes so that they are mapped into the same feature space. The calculations are as follows:

15 $${H}_{l\left(i\right)}={W}_{l\left(i\right)}\cdot {F}_{l\left(i\right)}$$

16 $${H}_{d(i)}={W}_{d(i)}\cdot {F}_{d(i)}$$

${H}_{l(i)}$ and ${H}_{d(i)}$ are the projected features of lncRNA node ${l}_{(i)}$ and disease node ${d}_{(i)}$, respectively. The three node feature dimensions are ultimately projected into a 64-dimensional feature space. ${w}_{l(i)}$ and ${w}_{d(i)}$ are the parameter weight matrices of the lncRNA and disease nodes, respectively, with dimensions of 240 × 64 and 412 × 64.

In homogeneous graphs, neighboring nodes exhibit different levels of importance in the task of learning node embeddings. The GAT is an effective tool for learning graph representations because it assigns different weights to neighboring nodes of the central node. In our model, the GAT is used to learn node representations. Feature weights are learned adaptively in subgraphs composed of different metapaths. This approach can fully exploit the information in the heterogeneous network. Specifically, for a given subgraph, the GAT uses an attention mechanism to learn the importance of different neighboring nodes to the target node, and then, for the central node, the features of the neighboring nodes are aggregated based on the calculated scores. For different homogeneous subgraphs, the degree of contribution ${a}_{uv}^{P}$ of a neighbor node v to a node can be calculated as follows:

17 $${\varphi }_{uv }^{G}=LeakyRelu\left({\left({\left({H}_{u}\right)}^{T}\cdot {H}_{v}\right)}_{G}\right)$$

18 $${a}_{uv}^{G}=softmax\left({\varphi }_{uv}^{G}\right)=\frac{{\text{exp}}\left({\varphi }_{uv}^{G}\right)}{{\sum }_{k\epsilon {v}^{G}}{\text{exp}}\left({\varphi }_{uv}^{G}\right)}$$

where G is the type of subgraph, u is the target node, and v is the neighbor node in the homogeneous subgraph G. LeakyReLU is a nonlinear activation function with a negative slope set to 0.2. ${v}^{G}$ denotes the set of nodes contained in subgraph G according to the subgraph. Finally, the obtained ownership values are normalized with the softmax function to obtain the final weight coefficients ${a}_{uv}^{G}$.

Subsequently, the features of all neighboring nodes v are computed and aggregated with the attention coefficients to update the features of the target node u ${Z}_{u}^{G}$:

19 $${Z}_{u}^{G}=\sigma \left(\sum_{v\epsilon {v}^{G}}{a}_{uv}^{G}\cdot {H}_{v}\right)$$

$\sigma$ represents the ELU activation function.

To enhance the model's ability to capture different levels of information, we introduced a multihead attention mechanism to extend the attention scores between nodes. The multihead attention mechanism is an improved attention mechanism that calculates the attention scores between nodes k times and uses the average value as the final score. The embedded feature ${Z}_{u}^{G}$ obtained after the internode attention mechanism is:

20 $${Z}_{u}^{G}=\frac{{\sum }_{1}^{K}\sigma \left(\sum_{v\epsilon {v}^{G}}{a}_{uv}^{G}\cdot {H}_{v}\right)}{k}$$

Considering that the embedding of a particular node can only reflect the semantic information of that node one-sidedly, to obtain a more comprehensive and adequate node embedding, we introduced an attention mechanism at the metapath semantic level to calculate the weights that the nodes receive under different subgraphs. Subsequently, the weights are aggregated with the corresponding neighboring nodes and then nonlinearly transformed. The average value of the node features after the nonlinear transformation was used as the contribution value of each metapath. Thus, the weights of nodes under a certain type of subgraph ${W}_{u}^{G}$ are calculated

21 $${W}_{u}^{G}=\frac{1}{\left|V\right|}\sum_{u\in V}{q}^{T}\cdot tanh\left({W}^{G}\cdot {Z}_{u}^{G}+b\right)$$

22 $${\omega }_{u}^{G}=\frac{{\text{exp}}\left({W}_{u}^{Gj}\right)}{{\sum }_{j=1}^{GN}{\text{exp}}\left({W}_{u}^{Gj}\right)}$$

where V is the total number of nodes under the subgraph adjacent to target node u, tanh is the activation function, ${q}^{T}$ is the trainable semantic layer attention vector with dimensions set to 128, and b is the bias vector. GN is the number of subgraphs of different nodes, and ${W}_{u}^{G}$ is the contribution of different subgraphs to the target node u. After semantic embedding, the final embedding obtained is defined as follows:

23 $${Z}_{u}=\sum_{i=1}^{GN}{\omega }_{u}^{Gi}\cdot {Z}_{u}^{Gi}$$

### Feature extraction based on homogeneous graphs

A heterogeneous graph constructed based on the correlation between nodes lacks information about nodes of the same type. To further capture the potential characteristics of the presence of same-type nodes, we defined metapaths $L\to L$ and $D\to D$ of the same type of node to construct both lncRNA and disease homogeneous graphs. The construction of the homology graph still requires the establishment of a neighborhood matrix between the nodes. We chose to use the KNN algorithm to construct the respective association matrices of lncRNAs and diseases. Moreover, the KNN algorithm makes predictions based on neighboring samples, and choosing the right number of samples can effectively eliminate the influence of noise.

Based on the comprehensive similarity obtained, the KNN algorithm was used to find the top k lncRNAs or diseases that were most similar to the ith lncRNA or disease, respectively, and assigned values of 1 and 0, respectively. Subsequently, we obtained the association matrices of lncRNAs or diseases with themselves, i.e., ${E}^{lnc-lnc}$ and ${E}^{dis-dis}$. Their assignment formulas are as follows:

24 $${E}_{ij}^{lnc-lnc}=\left\{\begin{array}{l}1\quad if\; j \in {Nei}_{li}\left(k\right)\\ 0\; otherwise\end{array}\right.$$

25 $$E_{ij}^{dis - dis} = \left\{ {\begin{array}{*{20}l} 1 & {if\; j \in Nei_{di} \left( k \right)} \\ 0 & {otherwise} \\ \end{array} } \right.$$

where ${Nei}_{li}(k)$, (${Nei}_{di}(k)$) contains the top k most similar lncRNA sequences (diseases) and lncRNA li (disease di) contains itself. We empirically set k to 20.

We defined the lncRNA homogeneous graph ${G}_{l}=(V,E)$ as containing the set of nodes ${v}^{lnc}$. The edge E in the graph can be defined as ${E}^{lnc-lnc}\in {R}^{{N}_{lnc}\times {N}_{lnc}}$, where ${N}_{lnc}$ denotes the number of lncRNAs in the dataset. Given lncRNA nodes ${l}_{i}$ $({l}_{i}\in {N}_{lnc})$ and ${l}_{j} ({l}_{j}\in {N}_{lnc})$, ${l}_{i}$ and ${l}_{j}$ are associated with each other if the association matrix ${E}_{ij}^{lnc-lnc}=1$. Additionally, we defined the disease homogeneous graph ${G}_{d}=(V,E)$ containing the set of nodes ${v}^{dis}$. The edge E in the graph can be defined as ${E}^{dis-dis}\in {R}^{{N}_{dis}\times {N}_{dis}}$, where ${N}_{dis}$ denotes the number of disease nodes in the dataset. Given disease nodes ${d}_{i}$ $({d}_{i}\in {N}_{dis})$ and ${d}_{j} ({d}_{j}\in {N}_{dis})$, if the association matrix ${E}_{ij}^{dis-dis}=1$, then there is an association between ${d}_{i}$ and ${d}_{j}$. Conversely, this means that no association is observed between the nodes.

Subsequently, we used the combined similarity of lncRNAs and diseases as the feature vector of the nodes. For the constructed homogeneous graphs, we similarly used the multihead attention mechanism to aggregate the node features and finally obtained the embedded features Z_O_.

### LDA prediction

We performed feature enhancement for the initial lncRNA and disease similarity using heterogeneous graph extraction of metapaths and homogeneous graph aggregation, respectively. We concatenated the resulting final embeddings and used a fully connected layer to reconstruct the lncRNA and disease features for the final prediction.

The predicted probabilities of lncRNA node i and disease node j are calculated as follows:

26 $${y}_{ij}=sigmoid\left(W\left({Z}_{li}+{Z}_{dj}\right)+b\right)$$

${y}_{ij}$ represents the association probability between the final predicted lncRNA li and the disease dj. Additionally, we created a loss function during the model training to quantify the discrepancy between the model's predicted value and the actual value. We then combined this function with the gradient descent approach to efficiently optimize the model's parameters and boost its predictive capability. The model uses an Adam optimizer for the gradient descent algorithm [33]. The following is the formula for calculating the loss function:

27 $$LOSS=-\left(y{\text{log}}{y}_{ij}+\left(1-y\right){\text{log}}\left(1-{y}_{ij}\right)\right)$$

y represents the true association of lncRNA with the disease. Finally, the model was trained by a backpropagation algorithm to obtain the final prediction probability.

### Comparison with other methods

To further validate the performance of the model, based on dataset 1, we compared the proposed method with five benchmark models. The BiGAN [28] is a generative adversarial model that consists of an encoder, a generator and a discriminator for predicting the associations of novel lncRNAs with diseases. HOPEXGB [34] is a prediction method based on machine learning techniques that uses higher order proximity preserving embedding (HOPE) and extreme gradient boosting (XGB) to identify miRNAs and lncRNAs associated with diseases. VGAELDA [35] is an end-to-end model that integrates variational inference and a graph autoencoder for lncRNA-disease association prediction. GCRFLDA [36] is a prediction method based on graph convolution matrix complementation. SIMCLDA [37] is a method for predicting potential lncRNA-disease associations based on inductive matrix complementation. GAMCLDA [15] is a method based on a graph self-encoder and matrix completion.

### Experimental setup

We used a fivefold cross-validation approach to evaluate the models. Our method is based on the PyTorch framework and executed with the dgl package. The computing environment included the Windows 10 operating system with an Intel(R) Core(TM) i5 and 16 GB of RAM. The maximum number of epochs in our model was 500, and all the trainable parameters were learned using the Adam optimizer with a learning rate of 0.001 and a weight decay rate of 0.005.

### Evaluation metrics

Referring to the evaluation metrics based on previous studies, we used the receiver operating characteristic (ROC) curve, precision, recall, and F1 score. Additionally, we used three other evaluation metrics, namely, accuracy, sensitivity, and the F1-score. These metrics were calculated as follows:

28 $$Accuracy=\frac{TN+TP}{TN+TP+FN+FP}$$

29 $$Sensitivity (Recall)=\frac{TP}{TP+FN}$$

30 $$F1-score=\frac{2\times \mathit{Pr}ecision\times Recall}{\mathit{Pr}ecision+\mathit{Re}call}$$

## Results

### Comparison with other advanced methods

As shown in Table 2, compared to the performance metrics of the benchmark model, MMHGAN's overall performance metrics are all higher than 88%. These results are better than those of GCRFLDA (86%), which is the best overall performing model among the benchmark models. MMHGAN has four evaluation metrics that are better than those GCRFLDA. However, the AUPR achieved by MMHGAN is lower than that of GCRFLDA. While the other models achieved good AUC/ACC performance, the performance in terms of the AUPR and recall was less than 80%.

**Table 2:** Comparison of different models

Model	AUC (%)	AUPR (%)	ACC (%)	Recall (%)	F1-score (%)
BiGAN	89.32	88.57	80.16	79.90	80.05
HOPEXGB	89.88	76.67	**99.34**	79.87	86.91
VGAELDA	91.26	76.58	97.18	40.97	58.63
GCRFLDA	95.48	**95.12**	88.59	86.89	87.55
SIMCLDA	84.33	88.24	75.49	89.97	78.59
GAMLDA	93.35	3.75	48.99	**93.64**	1.91
MMHGAN	**96.07**	93.23	89.43	89.03	**88.40**

### Model performance with different datasets

**Fig. 2:**
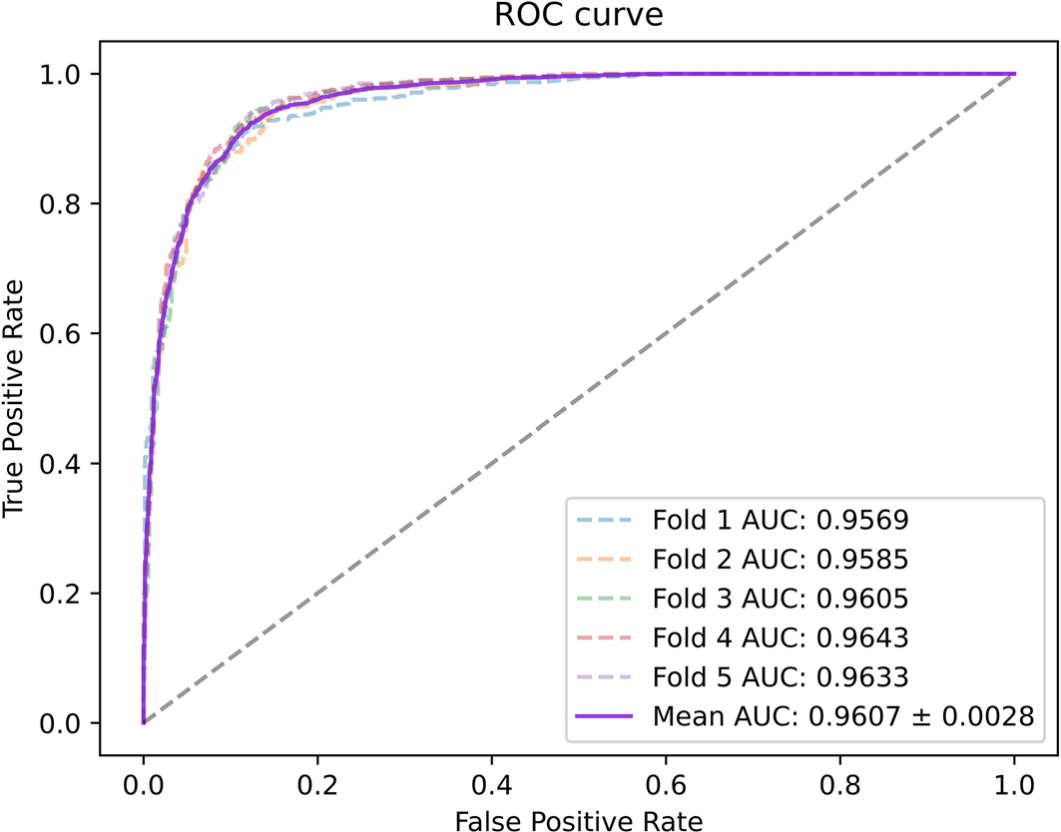
ROC curves generated by the MMHGAN model under fivefold-cv on dataset 1

**Fig. 3:**
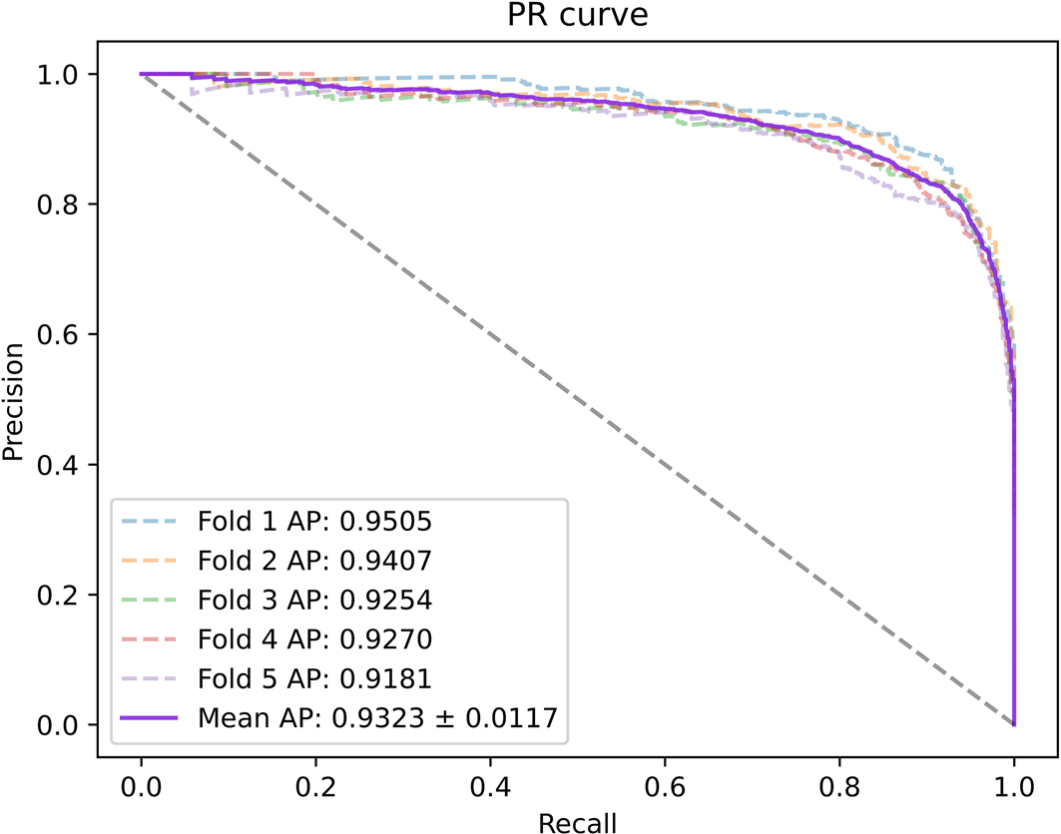
PR curves generated by the MMHGAN model under fivefold-cv on dataset 1

**Fig. 4:**
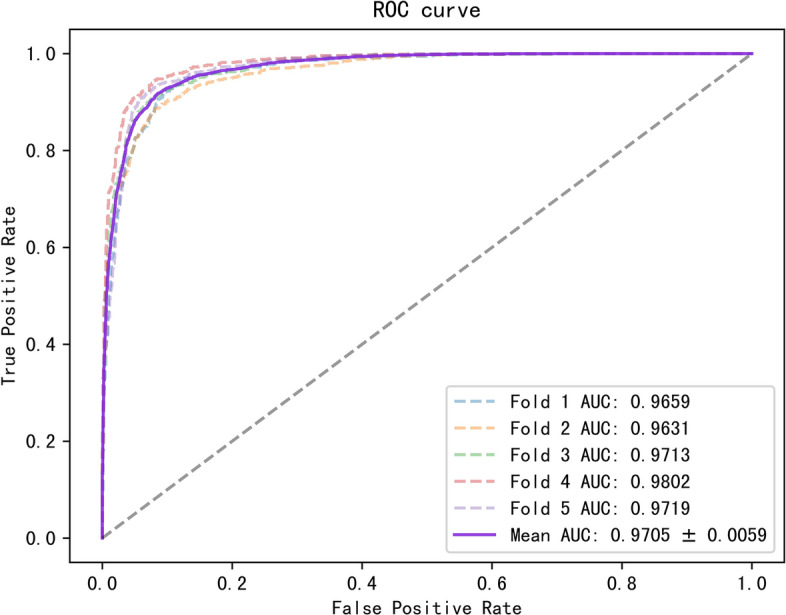
ROC curves generated by the MMHGAN model under fivefold-cv on dataset 2

**Fig. 5:**
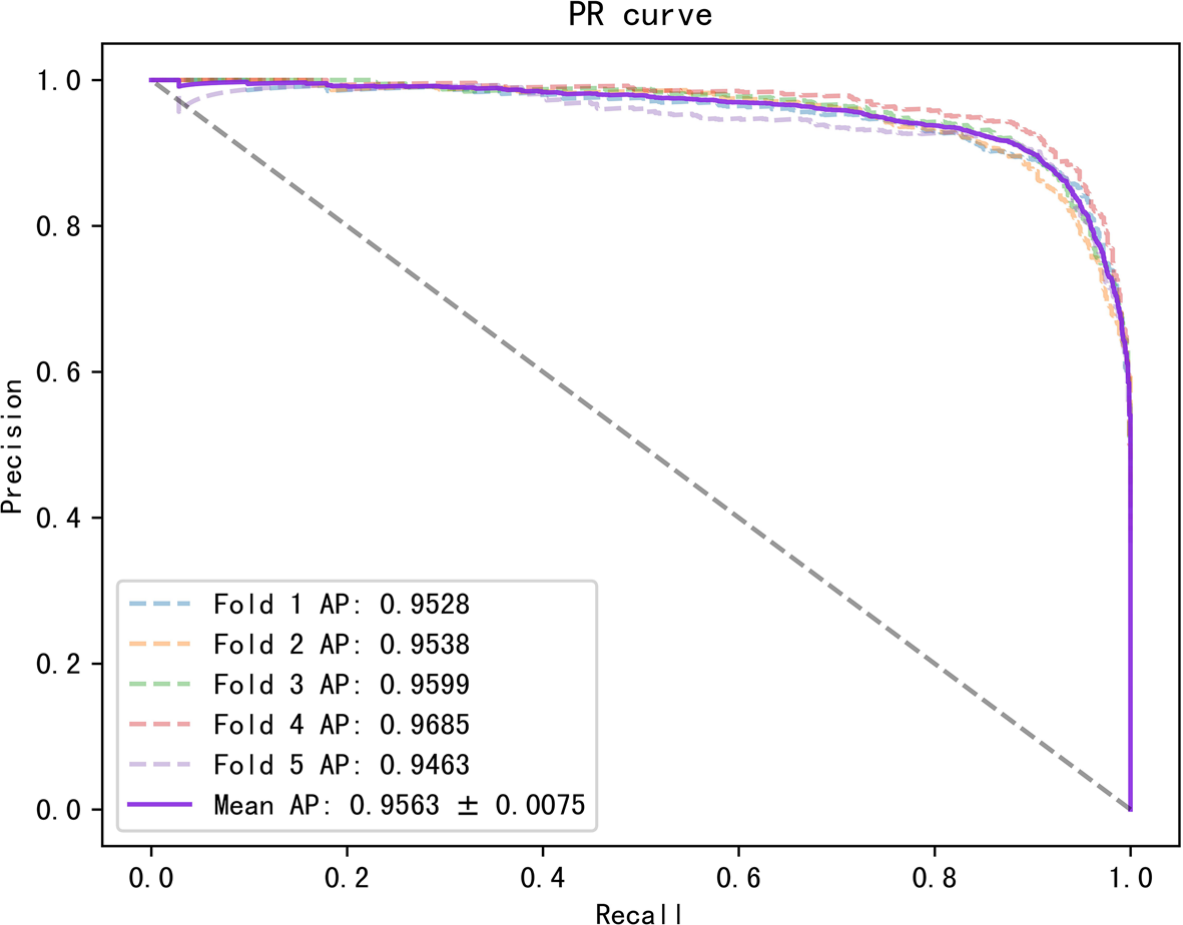
PR curves generated by the MMHGAN model under fivefold-cv on dataset 2

**Fig. 6:**
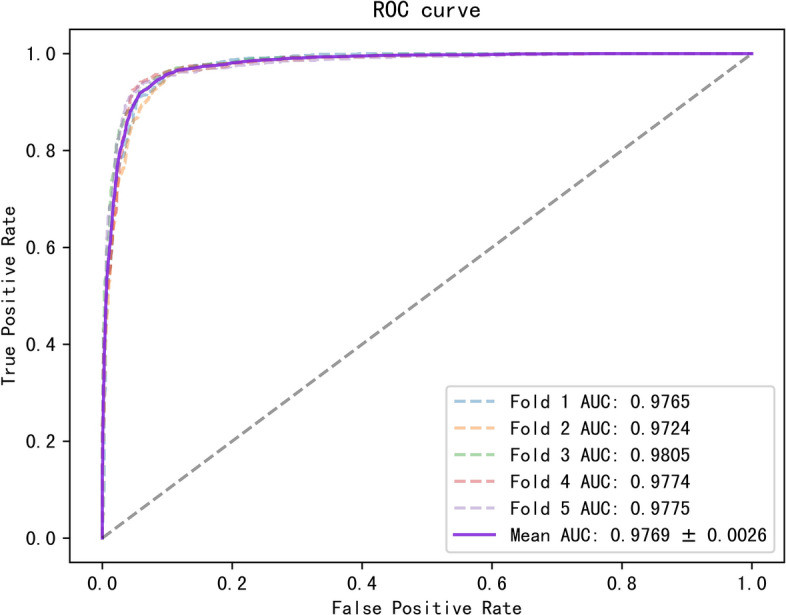
ROC curves generated by the MMHGAN model under fivefold-cv on dataset 3

**Fig. 7:**
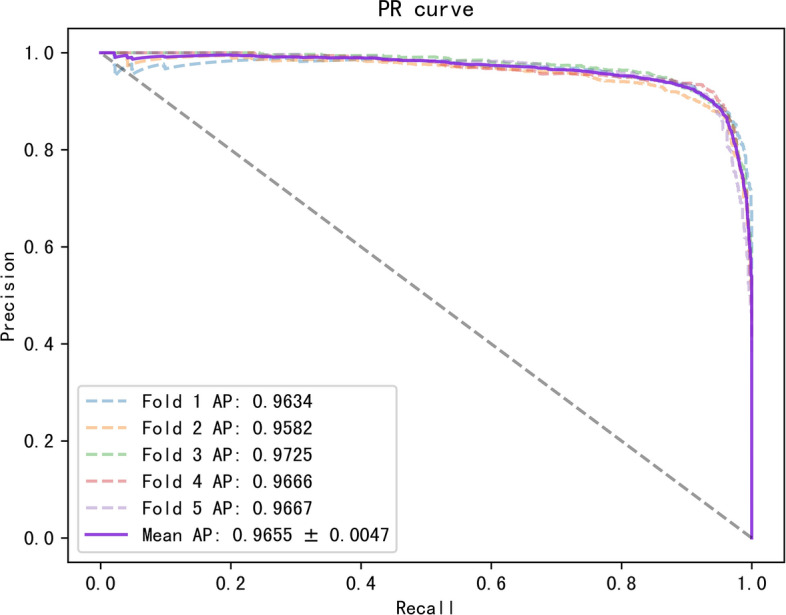
PR curves generated by the MMHGAN model under fivefold-cv on dataset 3

To better evaluate our model, we tested it on three datasets with multiple evaluation metrics, and the results are shown in Table 3. On these three datasets, all the metrics of the model were greater than 88%. The ROC and PR curves of our model on the three datasets are shown in Figs. 2, 3, 4, 5, 6, 7.

**Table 3:** Results for different datasets

Model	AUC (%)	AUPR (%)	ACC (%)	Precision (%)	Recall (%)	F1-score (%)
Dataset 1	96.07	93.23	89.43	89.84	89.03	88.40
Dataset 2	97.05	95.63	91.51	89.95	89.38	89.58
Dataset 3	97.69	96.55	92.32	91.10	92.14	91.62

### Ablation experiment

#### Comparison with different feature combinations

To further test the effect of different features on the classification results, we performed the following comparisons:

MMHGAN-NHO: This model aggregates node features only in heterogeneous graphs in the module identified as (iii) in Fig. 1.

MMHGAN-NA: For subgraphs obtained from different metapaths, in the module labeled (iii) in Fig. 1, we set the coefficient of the aggregated features of the subgraphs obtained through different nodes to 0.5 without weight assignment, i.e., the computation node of module (iii) labeled attention.

We compared these two models with the original model, and the comparison results are shown in Table 4. The results show that the model with richer feature information and more diverse attention mechanisms achieved better performance.

**Table 4:** Results for different features of the MMHGAN model

Model	AUC (%)	AUPR (%)	ACC (%)	Precision (%)	Recall (%)	F1-score (%)
MMHGAN-NHO	94.12	95.41	89.16	85.41	91.95	87.96
MMHGAN-NA	95.53	94.78	88.32	86.60	89.36	89.43
MMHGAN	96.07	93.23	89.43	89.84	89.03	88.40

### Analysis of parameters

**Fig. 8:**
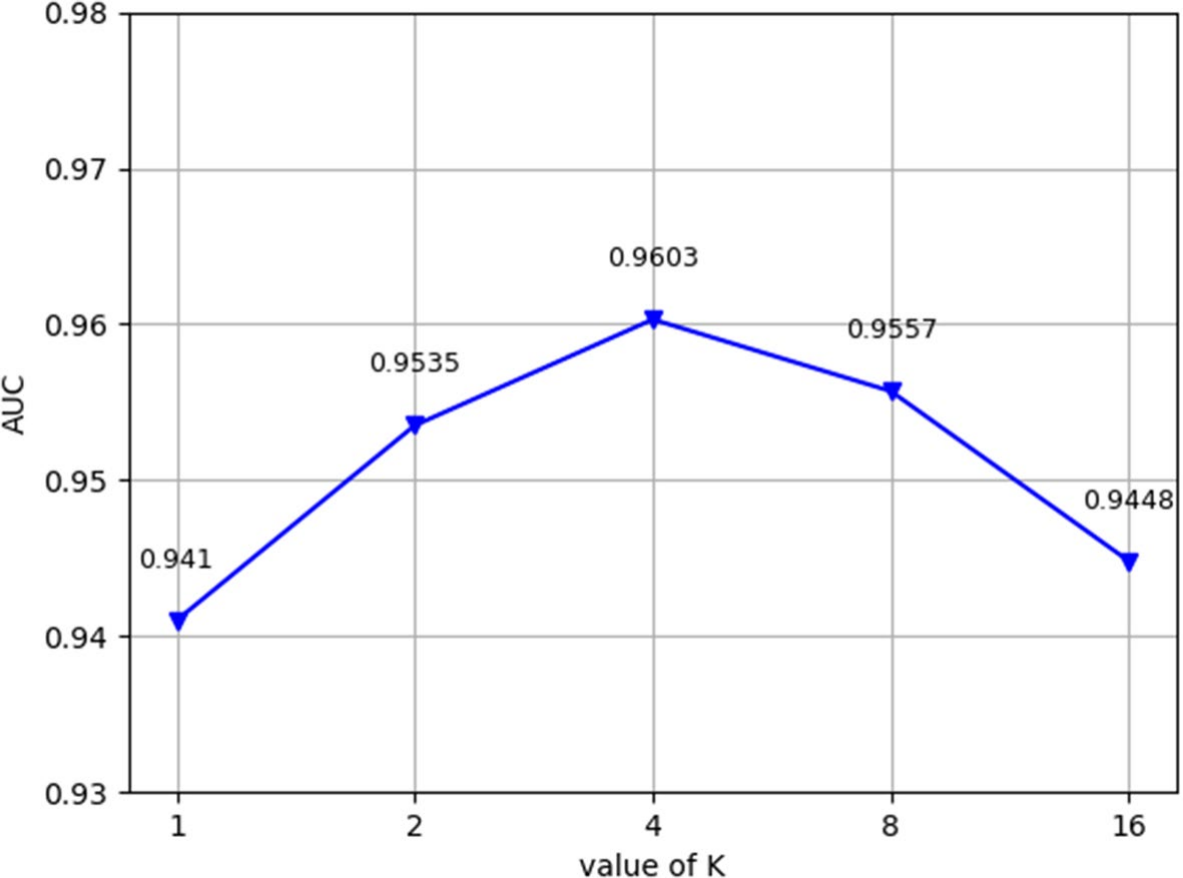
Model performance for different values of k

**Fig. 9:**
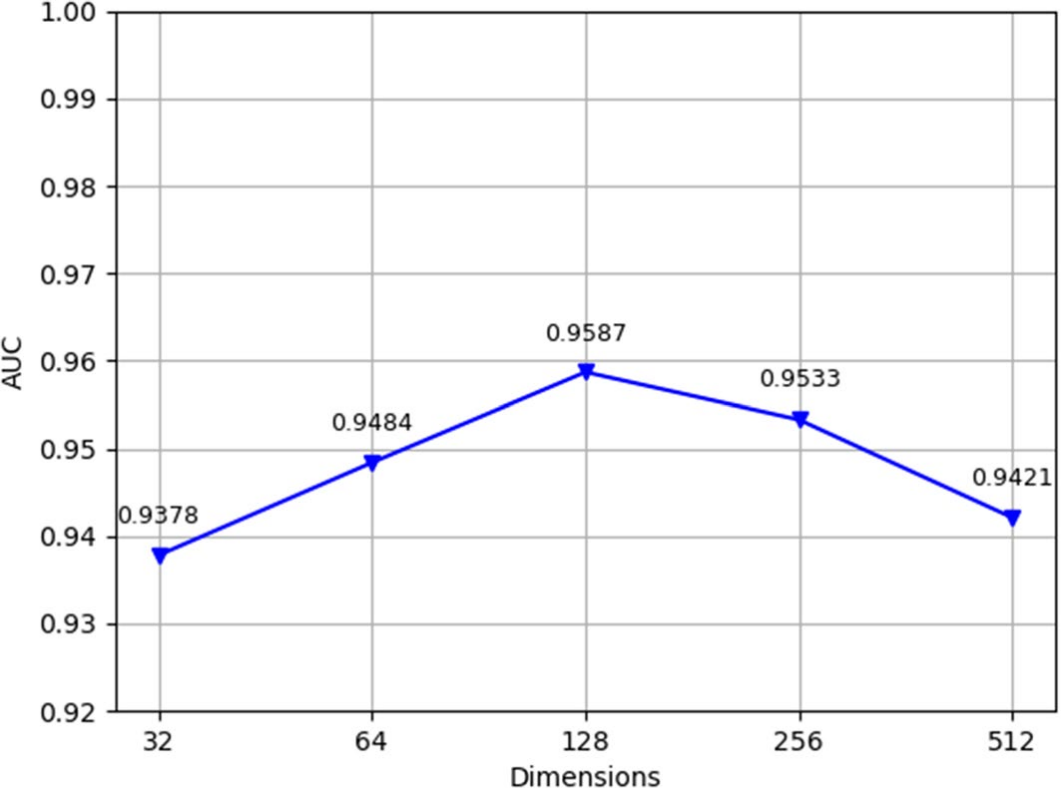
Dimensions of the output vector

By altering some of the parameters in this model, we can increase its performance. We assessed the value of k in the multiple attention mechanism first. We used k = 1, 2, 4, 8, and 16, and the resulting AUC findings are displayed in Fig. 8. As demonstrated, the model functions best when k = 4. The model is equivalent to that without the multiple attention mechanism when k = 1. The model effect was outperformed by the effects of other k values. This result demonstrates how the multihead attention method can be used to more fairly assign the weights of metapath instances. Second, we tested the different dimensional features of the attention layer and the output features, and Fig. 9 shows the AUC values of the MMHGAN model prediction results when the dimension n of the output features is different. It is clear that as the number of dimensions increases, the AUC value for the MMHGAN model increases. The model produces the best prediction results when the number of dimensions is 256. When there are more than 512 dimensions, the model's performance decreases, perhaps as a result of the model's increased propensity for overfitting, which yields subpar results. We therefore chose 128 as the number of dimensions.

### Case study

We studied three cases, lung cancer, esophageal cancer, and breast cancer cases, to further evaluate the performance of the model in predicting the associations between lncRNAs and diseases. For the studied diseases, we filtered out the associations between diseases and lncRNAs and constructed the same number of negative samples for training using the remaining associations between diseases and lncRNAs as positive samples. The diseases to be studied were subsequently entered into the trained model as test samples to obtain the prediction scores. We ranked the scores and selected the 15 lncRNAs with the highest scores as diseases with possible associations for the final predictions. For the prediction results, we compared the results by reviewing the LncRNADisease database, the Lnc2Cancer database, and the published literature. The final predictions for these three diseases are shown in Table 5, 6, and 7.

**Table 5:** The top 15 lung cancer-related lncRNA candidates

Rank	LncRNA name	Description	Rank	LncRNA name	Description
1	KCNQ1OT1	LncRNADisease	9	CDKN2B-AS1	LncRNADisease
2	MALAT1	LncRNADisease	10	MEG3	LncRNADisease
3	XIST	LncRNADisease	11	HOTTIP	LncRNADisease
4	H19	LncRNADisease	12	AFAP1-AS1	LncRNADisease
5	HOTAIR	LncRNADisease	13	PVT1	LncRNADisease
6	TUG1	LncRNADisease	14	BCYRN1	LncRNADisease
7	MIR17HG	Lnc2Cancer	15	HULC	literature
8	GAS5	LncRNADisease

**Table 6:** The top 15 esophageal carcinoma cancer-related lncRNA candidates

Rank	LncRNA name	Description	Rank	LncRNA name	Description
1	NEAT1	LncRNADisease	9	AFAP1-AS1	LncRNADisease
2	MALAT1	LncRNADisease	10	GAS5	Unknown
3	XIST	Lnc2Cancer	11	HOTTIP	Unknown
4	HOTAIR	LncRNADisease	12	MEG3	LncRNADisease
5	TUG1	LncRNADisease	13	PVT1	LncRNADisease
6	H19	LncRNADisease	14	HNF1A-AS1	LncRNADisease
7	MIR17HG	Unknown	15	BANCR	LncRNADisease
8	CDKN2B-AS1	LncRNADisease

**Table 7:** The top 15 breast cancer-related lncRNA candidates

Rank	LncRNA name	Description	Rank	LncRNA name	Description
1	KCNQ1OT1	LncRNADisease	9	CDKN2B-AS1	LncRNADisease
2	NEAT1	LncRNADisease	10	GAS5	LncRNADisease
3	MALAT1	LncRNADisease	11	CASC2	LncRNADisease
4	XIST	LncRNADisease	12	AFAP1-AS1	LncRNADisease
5	H19	LncRNADisease	13	MEG3	LncRNADisease
6	HOTAIR	LncRNADisease	14	HOTTIP	Unknown
7	TUG1	LncRNADisease	15	PVT1	LncRNADisease
8	MIR17HG	LncRNADisease			

Lung cancer is a malignant tumor originating from lung tissue cells that usually spreads through the respiratory tract and is associated with extremely high morbidity and mortality. The prediction results confirmed the presence of all the predicted lncRNAs. The results suggest that the lncRNAs predicted by the model are indeed associated with lung cancer.

Esophageal carcinoma is one of the most common tumors of the digestive tract. Therefore, we chose it as the second case to test the model. Table 6 shows that the predicted associations of 12 of these lncRNAs with diseases can be retrieved from the LncRNADisease and Lnc2Cancer databases.

Breast cancer was studied as the third case. Breast cancer is one of the most common malignant tumors in women and originates from breast epithelial or ductal cells. Its incidence increases with age. As shown in Table 7, 14 of the 15 predicted lncRNAs were confirmed by databases such as lncRNADisease. The above three case studies demonstrated the ability of the MMHGAN model to predict potential lncRNA-disease associations.

### KM curve

A Kaplan–Meier curve is a statistical tool used in survival analysis, usually to describe the probability of an event occurring within a certain period. Survival analyses are primarily used to study the time to the occurrence of an event, which can be the onset of a disease, death, or other specific outcome.

Survival time ${t}_{i}$ is the horizontal coordinate, and survival rate ${S}_{{t}_{i}}$ at each time point is the vertical coordinate; the continuous curve formed by connecting the survival rates at each time point is referred to as the survival curve.

**Fig. 10:**
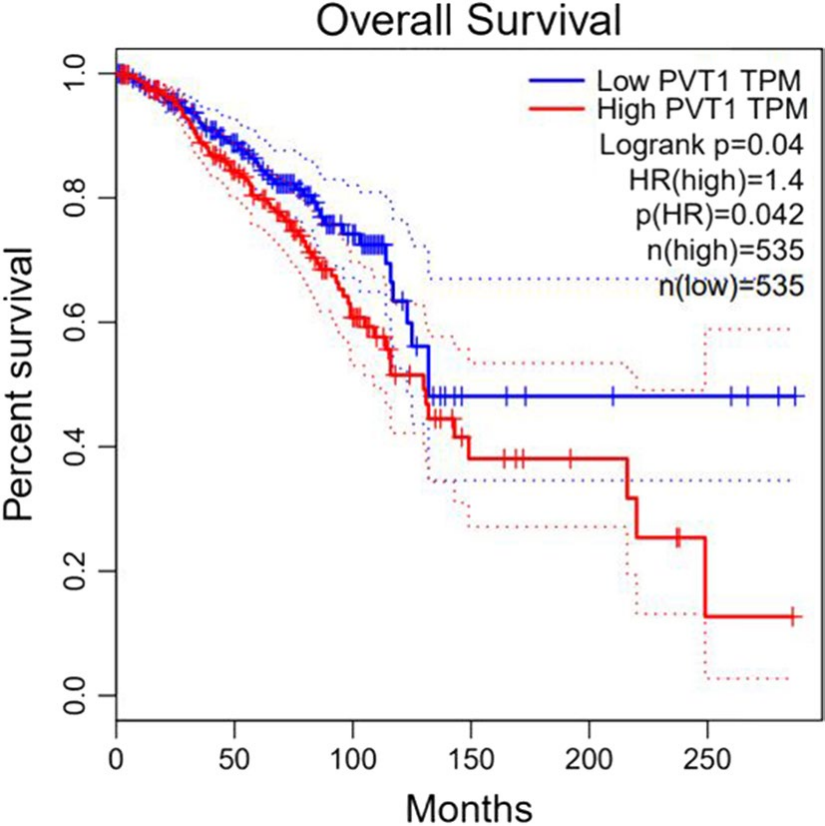
Survival analysis of breast cancer patients with PVT1

**Fig. 11:**
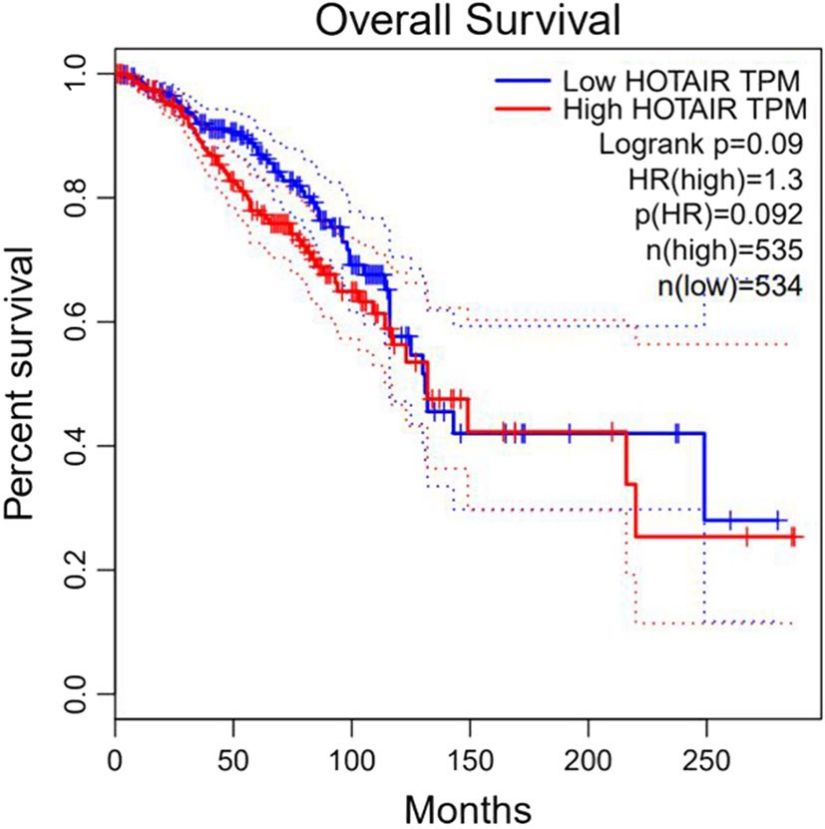
Survival analysis of breast cancer patients with HOTAIR

Based on the results of the case study, we selected breast cancer for survival analysis based on TCGA [38] data. As shown in Fig. 10 and Fig. 11, for PVT1 and HOTAIR, the survival rates of patients with low lncRNA expression are higher over time.

## Discussion

To make full use of lncRNA and disease intermediate information to enhance LDA prediction, we proposed the MMHGAN model to learn each homogeneous graph or heterogeneous subgraph of a specific metapath using a GAT network. In addition, we used the KNN algorithm to construct homogeneous graphs and used an attention mechanism to adaptively assign weights to different heterogeneous metapath subgraphs to achieve denoising and to obtain additional semantic information. The cross-validation results show that the overall performance of the model outperforms that of the baseline comparison method.

Several studies have been conducted to introduce primary and deeper information for disease association prediction through the k-nearest neighbors (KNN) algorithm, and the model performance has further improved. These studies have validated the effectiveness of combining the KNN algorithm and GCN in disease association prediction. Consistent with these studies, we also constructed homogeneous subgraphs using the KNN algorithm and acquired features using the GAT. The difference is that our homogeneous graphs in the input KNN algorithm are the LSM and DSM, which are the merged similarity matrices of lncRNAs and diseases after linear fusion.

To explore better disease association prediction models, different approaches have been used to fully exploit disease association information. Yang [28] et al. introduced the generative anti-network approach to lncRNA disease association prediction. Shi [35] et al. proposed VGAELDA, which integrates variational inference and a graph autoencoder through the integration of graph representation learning and alternating training involving variational inference, which enhances the ability of VGAELDA to capture efficient low-dimensional representations from high-dimensional features. Fan [36] et al. proposed GCRFLDA, a prediction method based on graph convolutional matrix complementation. utilizing conditional random fields and attention mechanisms to form encoders and decoders, learn efficient embedding of nodes, and score lncRNA-disease associations. As shown in Table 2, although these methods use different techniques and obtain good performance (AUC > 89%), they do account for the rich semantic information in heterogeneous graphs. He [34] et al. proposed a prediction method based on machine learning techniques to identify disease-related miRNAs and lncRNAs by higher-order proximity-preserving embedding (HOPE) and extreme gradient lifting (XGB) using a heterogeneous disease–miRNA‒lncRNA (DML) information network. Lu [37] et al. proposed a prediction method based on disease–gene and gene–gene correlations, computed the Gaussian interaction spectrum kernel of lncRNAs, and proposed a method to predict potential lncRNA-disease associations on the basis of inductive matrix complementation. Wu [15] introduced graph self-encoders to learn lncRNAs and characterize diseases through their ability to encode and decode graph structures and features. While these methods have advanced the field by considering heterogeneous graph-rich information, they have not fully exploited the potential of heterogeneous graph-rich information, as shown in Table 2, where the overall performance of the methods was 75%. In addition, these methods do not further consider the information of the intermediate nodes of the metapath subgraph. Inspired by Xuan [16] and Zhao [17] et al., we utilized subgraphs constructed from homogeneous graphs and heterogeneous graphs as inputs and adopted multipath subgraphs combined with a multihead attention mechanism to acquire features, fully considering the information of the intermediate nodes of the metapath subgraphs. As shown in Table 2, our method's AUC, ACC, recall, and F1 score are 0.59%, 0.48%, 2.05%, and 0.85% greater than those of the best baseline model, GCRFLDA.

Our study is inspired by GSMV, a new association prediction model proposed by Xuan et al., and HGATLDA, a novel metapath-based heterogeneous graph attention network framework developed by Zhao et al. Unlike the HGATLDA approach, these methods do not consider homogeneous subgraph information. We obtained the features of homogeneous subgraphs through a multihead attention mechanism; in addition, unlike GSMV, which uses metapath instances to obtain semantic information, we used metapath subgraphs to obtain semantic information. Subgraphs can better capture local structural information and are more interpretable; additionally, when dealing with sparse matrices, metapath extraction of subgraphs can reduce the computational complexity and noise interference, and it is easier to adapt to different requirements and data characteristics by extracting subgraphs according to different paths.

As shown in Table 3, our model performs better on dataset 2 and dataset 3 than on dataset 1, which may be due to the different data sample sizes.

Despite the good results of our model, there are still several limitations. First, there was an imbalance of positive and negative samples in the datasets; for example, in the first dataset, only 2697 associations existed between 240 lncRNA nodes and 412 disease nodes, which was insufficient for predicting the results. Second, generating subgraphs was used in the model to aggregate the features, and the complexity of the model increased when the amount of data increased. In addition, we did not validate the results predicted by the model through biological experiments; in the future, we will add biological wet experiments to further evaluate the model's performance.

## Conclusion

In this paper, we proposed a hierarchical network model of multiple metapaths, MMHGAN, to extract features from a multiview perspective and to mine the semantic information contained in different graphs for predicting potential lncRNA-disease associations. By constructing both homogeneous and heterogeneous graphs, the information provided by the neighboring nodes of lncRNAs or disease nodes can be mined more comprehensively. In addition to the KNN algorithm and the method of constructing subgraphs through metapaths, the noise generated by sparse matrices can be effectively reduced, which can lead to better performance of our model. Moreover, we introduced miRNA nodes to construct a ternary heterogeneous graph. To better explore the structural information provided by the heterogeneous graph, we generated corresponding subgraphs with the help of different nodes and used the GAT network to enhance the features. We assigned different weights to the subgraphs constructed by different nodes to obtain more semantic information. Finally, the MMHGAN also outperforms the other methods. In the case study, the capability of the MMHGAN model is further confirmed.

## Data Availability

The data and code can be downloaded from the following website: https://github.com/ydkvictory/MMHGAN.
